# A Case of Monomania

**Published:** 1848-10-01

**Authors:** 


### A CASE OF MONOMANIA, WITH REMARKS.

A MAN may receive a blow upon his heart or his imagination as he may receive an injury to his brain, which till, if ever, the effects have passed away, shall determine an eccentricity of conduct in all after-life, for which he is wholly, or in a considerable degree, irresponsible. Such a man is *pro tanto* to be pitied, and, if needful, shut up. If his eccentricity be of dangerous tendency, society is to be protected from him; he is to be protected from himself. An accidental occurrence impressing its image upon a susceptible imagination may spread a blight over the mind and cripple its capacities, so that on some one subject, directly—on others bearing affinity to it, implicatively—its views shall be far otherwise than sane. Let it be proved that in any man's case such a condition of mind shall during a great portion of a lifetime have existed, concealed by the force of a strong will strenuously exerted with success to conceal it, and such condition consequently have continued, and a lesson of much moral utility may be learned from it. If, especially, it were found that, owing to such a condition of mind, a man had acquired not a bad character only, but a character, bad or good, the reverse of his real character, it would supply a touching illustration of the Divine wisdom of the Scriptural injunction to "*Judge not at all.*" There may have been insanity hiding itself under the mask of vice, the spectacle of a man deliberately and of set purpose damaging and encouraging damage to his own character. There may have been the affectation of frailties foreign from and abhorrent to his nature: there may be lost character—ruined fortunes. All may find a climax in a death resembling that of the Spartan boy who suffered the fox to tear out his bowels. He may face not only certain adversity here, but foreknown infamy after death, when but a few words said would clear his reputation from every cloud. He may have been only a victim and a martyr. He may have preserved the appearance of sanity at the expense, perhaps by the total sacrifice, of character. It is, indeed, by no means uncommon that a man should less like to be thought weak than wicked. In so far as the mind is sane, this is itself wickedness: it is fearing man more than God. Upon this point, collective man—society—judging unlike the Almighty, misjudges. But such is the judgment of man. To be intellectually weak, if inculpably and unconsciously, may be to be despised, but it is not to be wicked: it is only moral weakness that is wickedness. We do not speak here, however, of the errors in morals of a sane mind, but of a mind which has sustained some shock which has crushed or greatly impaired its powers, or some one or more of its powers. It is true that there are various shades of insanity; that there are cases in which it lies scarcely within the compass of human judgment to pronounce where moral responsibility ends and moral irresponsibility commences. It is of a case of a sufficiently marked character that I am about to speak. Yet during the greater part of a lifetime nothing overt was exhibited by which to discriminate its real character, and much that was calculated to mask it not from observation only, but from suspicion. Having thus introduced the case, on which I set the more value from an impression that it is typical of a class of cases not in main points dissimilar, I shall draw no forced inferences from it, leaving many of such reflections as will spring naturally from it to occur to the mind of the reader. I only think it necessary here to add that I have the patient's free leave to communicate it for the benefit of the profession and of society, and his fervent wishes that it may tend to the benefit of both. Excepting for the sake of a decent circumlocution, no technical phrases are used, none being required.

Some cases are partly or wholly physical, requiring therapeutic treatment: this was wholly mental, requiring psychological treatment. It received no treatment at all: time and the current of life and of events cured it, but cured it too slowly and too late. Of the overt in this person's character and conduct it may be said that he once attempted self-destruction, but failed: that no jury, had he died, could, according to evidence adduced, have well brought it in otherwise than as *felo-de-se:* that his mental faculties both before and after this appeared sound and strong; but that evidences of penitence were small, though the attempt was never repeated: that he filled well situations of high responsibility, requiring both bodily and mental exertion; but that he acquired also a reputation for occasional outbursts of profligacy and debauchery. This ill repute he, past all question, merited; yet did it seem as if there were a want of consistency between these vices and failings and the general tenour of his character; such a want of verisimilitude, in short, as we should ill tolerate in a work of fiction. The key to this mystery remained long in his own sole possession; not for years was it surrendered to the knowledge and keeping of any second person. During these long years he was a conscious monomaniac. How much misery may be told in a few words! The true history of his case may be briefly narrated as follows:—At the age of about seventeen, a case of real, or more likely imaginary, impotency came to his knowledge, and the contempt and ridicule with which it was treated by his informant shocked him unspeakably. "Certainly," he thought and felt, and it was the reasoning of a correct mind and the sentiment of an honest heart—"a man so unfortunately circumstanced should meet with everything from society, and from his trusted friends especially, that could be compensative to him for a condition so deplorable: we do not mock at the blind or the deaf—why, then, at him? See, however, how he is treated! What an aggravation to an already dreadful fate!" He thought and felt this, but did not say it. "If I utter a syllable in check of this torrent of ridicule," he thought, "I shall be suspected, as a matter of course, to be in the same predicament; and to such contumely as that to which he seems universally obnoxious, death would be preferable." It did not stop here. "What," he thought, "if I should really be in the same predicament; perhaps I am? There are doubtless many who are—why should not I be one of them? See with what advertisements for the cure of such every newspaper is crowded." The impression that he might belong to this numerous and unhappy class passed through various gradations of dread, till a sense of scepticism merged into an almost belief that it was so with him. Such was his horror at the contemptuous comments which cases of this nature incurred, that, coupled with his entertainment of this false and fatal chimera was a vow which he made and kept, never to make any human being his *confidant* on such a subject, but at least to pass through life and perish in silence. But it was a doubt, and not a certainty—and then to know whether it was so or not—that was the thing. It might be easily ascertained, and to live in suspense upon this dreadful point—oh, it was intolerable! And then there was an assignation, unprompted by sentiment or passion, but by a horrible dread lest the issue of the experiment should prove unpropitious, and that issue was what might have been expected: and there was, to his imagination, the absolute confirmation of his direst apprehensions. And then, why should he live—who would live? it would be the kindest thing he could do towards his parents—his sisters—his family—to put himself out of the way: and he attempted this, and not half-heartedly, though he failed. And then, recovering, he still—so cogent was his secret madness—not only still kept his vow made to himself—but rejoiced at, almost countenanced, surmises on the part of others of the cause which impelled him to the attempt which compromised his probity—for such surmises proved that the truth was not suspected. But though he knew that he was mad, and not master of himself when he made the attempt upon his life, and felt that it would be other than fair to accuse himself of it as a crime, whereas it was the result of involuntary distraction, and overthrow of reason, he inferred that, the attempt not having succeeded, it was not the will of God that it should succeed; and resolved—a resolution which, like the first-mentioned one, he sacredly kept—never to repeat it. But the supposed cause still existed, and he sought to reconcile himself to his fate. He never seemed otherwise than sane—the attempt itself excepted—either before or after: he expressed neither sorrow nor repentance; but he sought no less consolation in religion as the only source of consolation left to a person in his supposed condition. He turned to his Bible, and he read in Isaiah—"Let not the eunuch say I am a dry tree," and "God will be better to him than sons and daughters." And he perceived that the Almighty did not treat with contumely persons of this class, and thought that surely then neither ought man: but then he thought also that whatever man ought to do as to this, he does not do as he ought, but as he ought not; and he kept faithfully, therefore, still his own counsel. Rather far than that the supposed truth should be suspected, he would have suffered himself to be innocently sentenced to transportation for felony or be hanged for murder.

All this while—that is, during some years—he was not only, apart from this weak point in his brain, as "simple as a child," and with "no more harm in him than a mouse," but competent to the vigorous pursuit of severe studies, and the efficient fulfilment of arduous duties. He was accustomed, in his conversations with me, to compare this shock received from the spectacle of the person pointed out to him as impotent, and from the contemptuous manner in which that person was spoken of—to the effect upon a shoemaker residing opposite the gate to which a nobleman was fixed alive, in '98, by the pikes of the insurgents, from the spectacle of his assassination, and of whom it is recorded that the horror of the event shocked him into a state of incurable idiocy. Was there the least foundation other than the shock occasioned by the event so made known to him for his suspicions of his own condition? None; nay, minus none. It was clear and sheer monomania, and nothing else, even fractionally, was admixed with it. There was nothing of real on which to base the imaginary. Now, it need not be said that, there being nothing real in it, this state of things would, life continuing, proceed to effect of itself some sort of cure. But fresh visions of the same Medusa's head—of the same horror—at first frequently, long occasionally, occurred. And there were the appearances in him of fickleness and perfidy towards the well-charactered of the opposite sex, towards whom he paid attentions, as of profligacy and libertinism towards those of easier virtue. He could not wholly put out of his sight and memory that horror, which, having once dethroned his reason, never ceased wholly from asserting its predominance over it; although it was not eventually in vain that his strength of will competed with it. But while the contest lasted, how much of life was wearing away!—what energies were being wasted!—what chances of fortune thrown away!—what heart-affections thwarted, in making war upon this shadow!—what sacrifice of character! —what submission to censures unmerited!—how was disrepute even courted by him!—how, even yet, through good and ill report, in so far as he was a free and responsible moral agent, did he act with honour!—how, reasoning upon false premises, did he nevertheless act as if the purpose of his life had been to distract and break the hearts of others and his own! Well, he has now a group of children round him—as the nurse in one of Beaumont and Fletcher's plays says, "as like him as if he had spit them;" or, as he himself averred, all this would have remained unknown, and he would have died unshriven of the confessions herein embodied. That his moral sense remains confused in some degree yet, is certain. Remembering the horrors of the past in his life, he speaks of communism and socialism, of anything and everything as being preferable to the sufferings he underwent, and then retracts his words; but never farther than to own that it is likely that such sufferings should have left his mind in a state of incapacity to judge of such matters rightly. There is, in short, a lasting confusion of mind upon this one topic. One feature is conspicuous, and that is, a boundless charity in judging either gently or not at all of the conduct of others in all matters of the heart and passions. There is nothing eccentric, little that was or is perverse in his temper, or wrong in his conduct, to which the shock his mind had sustained does not supply the one sole key. Effects often remain after causes have ceased to operate.

It is that power which a knowledge of the human heart gives, not craft and cunning, that is requisite in the treatment of the insane. It is not sufficient to be conversant with the best current professional works on insanity. The writings of Shakspeare and of Beaumont and Fletcher, and of our elder English dramatists, should be also studied; even in studying the latter, we but study nature at second hand; but in studying them, we do not study pictures copied from pictures, but well-drawn representations of realities,—many of their dramas display an accurate knowledge of some of the finer shades of insanity. There are lessons which we have yet to learn in the moral treatment of the insane, wherein they may be susceptible of moral treatment, which they are not always. The foregoing case was one admitting of such treatment, if it had been accurately fathomed. Years of bootless mental agonies might have been spared by the confidence of the party having been early obtained by a professional adviser, worthy of such confidence, capable of administering kindly and judiciously to the "mind diseased," incapable of abusing the confidence bestowed. It should be ever held in view as a truth, that to mental alienation and confusion, as certainly as to toothache, or any other bodily ailment, we are all alike, as human beings, liable ; that, otherwise than conventionally, and under the guidance of false estimates of men and things, no more disgrace is attached to any species of insanity than to an attack of rheumatism or small-pox. It is moral turpitude alone that degrades and disgraces,—not any one form of disease, mental or bodily, more than any other. To misprisions upon matters of faith and matters of the heart and passions, all are the more especially liable, because by these our nature is most moved—in these takes the deepest interest. It says nothing against religion or love, both of them features of our nature, and parts of our very being, that this should be the case. The finest-toned minds are not less prone "to jangle like sweet bells out of tune," than the coarse, the simple, and the unidead. Perhaps they are more so, as the most complicated instruments have a greater number of chords, any of which may be relaxed or over-strung into discord. Such are, at any rate, the best worth tuning and keeping in tune. Not the physician's skill only in the discrimination of mental disease, but the poet's penetration into the mysteries of the human heart, is necessary to render a man a truly accomplished practitioner. He should be wanting in no one high quality of the possession of which human nature is capable. For the absence of these loftier qualities, no aggregate of the smaller capabilities can compensate. Those capabilities are most little and low of stature which prompt their possessor to sneer at religion, because any certain proportion of the insane are what is called religious-mad. As well might they sneer at that master-passion of our nature, which, despite all ridicule, will ever co-exist with it. It is rather to be marvelled at, that considerations of the momentous interests which religion involves do not invariably—as the rule and not as the exception—unsteady the strongest minds. The minds of many whom they have actually unsteadied have been indisputably strong. For men to be only the safer from this species of insanity, in proportion as they are less capable of religion than others, is no great boast. It is as if the deaf should glory in their insensibility to the charms of music, or the blind to the beauties of nature, upon the ground of their insusceptibility to annoyance from the neighbourhood of discordant sounds, or the presence of disagreeable sights.

> " Should o'er the page inspired too closely pore
> The student, smit with love of sacred lore,
> Shall we, turned atheists, our creeds unsay,
> Because his overwearied brain gives way ?

All human hearts must passion cease to move,
Because some maiden drowns herself for love?
All the less scoffed at, as an empty dream,
Shall be for such mishap the engrossing theme."

There is nothing of the knowledge of which—nay, nothing scarcely of the belief of which—the professional attendant on the insane should not be capable. In his heart and brain should exist the images of all that is possible; for in dealing with the insane he has to deal with persons in whose hearts and brains are the images of much that is unlikely. He must comprehend the chaos of the deranged mind, or his chance of reducing it into order will be small. He must know both the true and the false, in order to trace the line of demarcation which divides them. He cannot know too much. He must precursorily know the true, or he can form no judgment with respect to the false. The first elements of human nature lie naked and in confusion before him; an acquaintance with the superficial, and the merely conventional, will avail him nothing. He will meet with pretensions to a sort of madness different from that which prevails within. He will have to encounter anomalies the most startling and incredible,—misprisions, whose very simplicity, if ascertained, will astonish him. Amid much that is irremediable, he will meet with much that can be remedied. The vista before him is wide and varied.

I am fully aware of the prominence lately given to spermatorrhea, as a disease which is the *origo mali* of much mischief. That it is a complaint, and a curable one, there is no doubt; that the instances of it are numerous, I have much doubt. As to spermatorrhea in the case recorded, there was no symptom of it; there were no nocturnal or otherwise casual involuntary emissions, no urethral discharge, simulating gleet. As to masturbation—that or some other *ation* is pretty general. I am inclined to think, the less said about it almost the better. Too much is said of it, when an empirical sway over the imagination and purse is usurped by the pamphleteer or the quack advertiser, through rhetorical flourishes and exaggerated narratives of cases.

It is against grace to lead a life of fornication and debauchery; but there is no disputing that it is against both grace and nature to indulge in this vicious propensity. Insanity, sometimes the cause, is sometimes the consequence of it, doubtless; but that there is much of the false and artful in appeals from quack practitioners to the public, is manifest. Where there is one case of true spermatorrhea, there are hundreds of cases of deranged or impaired functions, which neither begin, proceed, nor end in this evil propensity. I question whether spermatorrhea, where it exists, has anything to do with masturbation; whether it is not a complaint *per se* arising irrespectively of masturbation. In the only marked case I know thoroughly well, I will venture to assert that masturbation had nothing to do with it. There is much to be done by caustic in spermatorrhea; but in cases of this vice moral suasion and enlightenment of mind are the only remedies. We know that passions repressed from religious conviction, or from whatever cause, occasion much trouble; we have read of St. Jerome's tribulation on this account, and of his wife of snow; we read in Scripture of the existence of a class of persons, of whom St. Paul says, "it is better to marry than to burn;" we have read the heathen assertion—

"Expellas naturam furca, tamen usque recurrat."

The truth—and all the truth we can come at—will be arrived at through calculating the mean of a variety of errors. Virtuous efforts to do what cannot be done may lead to vice; thus it is that extremes are often seen to meet. But if we are to judge of human failings leniently, in proportion to their frequency, and as we should ourselves wish to be judged, most certainly the errors into which the master-appetites and passions of our nature betray us should be regarded with more compassion and allowance than any. I make no doubt, that it is in seeking to be fit company for saints and angels, in aiming at high moral excellence, that many have sunk into evil habits, which have placed them below the moral level of the beasts that perish. Still, there remain to be commended high original motives; there are allowances to be made for lapses into evil upon the ground of human frailty, and no small admixture of ignorance. Severe moral conflicts with physical forces, too strong to compete with, may have preceded and have led to habits in comparison with which known and overt vice is more respectable. In reasoning correctly on this subject, we shall always find ourselves reasoning in a circle whose boundaries we cannot overpass, nor stray away from.

Shakspeare says that

"Flowers which fester smell far worse than weeds;'

and, as an assertion in reference to human morals, it is said correctly, although, as a fact in vegetable physiology, Prout and Liebig might call it in dispute. Were it not melancholy, it would be ridiculous to follow the career of a man aiming at the gift of continence as the chief feature of a virtuous life, then lapsing into the bathos of masturbation, losing his health or being frightened into suspicions of his virility, and eventually at the mercy of the quack advertiser, and an object of compassion to the less low-fallen street-walker, seeking to possess himself of the—gift of incontinence; presenting the spectacle, not of a ruined frame so much as of a ruined mind. There are certain conclusions to be drawn, among which is one in chief—namely, that there are some men, constitutionally speaking, who ought to be married, or even to do worse, rather than do what is worse than that worse. A state of celibacy, after the age of puberty, is in truth, in all human beings of both sexes, an unnatural state—it is natural to none; to many, a pure, and undepraved, and happy state of celibacy is absolutely an impossibility. To the pernicious gratifications of the solitary vice alluded to, the indulgence of promiscuous sexual intercourse is in the next degree unnatural. There may be pleasures, but there is not happiness in any other sexual intercourse than that which is based on individual attachment. As to unnatural gratifications, they begin, go on, and end in certain unhappiness. Nature herself resents them; but they do not necessarily, if at all, and if ever incidentally, produce true spermatorrhea, which may be cured by caustic, but which is not necessarily a fair subject for caustic comments.

That spermatorrhea may exist as a complaint *per se* I am perfectly certain, and this to an extent which would render procreation unlikely, and masturbation itself scarcely practicable, seminal ejaculation taking place upon such slight provocation as to nullify the practice of either. When there is an indulgence in masturbation, there is not impotence; where it occurs in excess there is a strong *argumentum baculinum* against celibacy—there is evidence that the war with nature has been waged too long, and that it is time that there should be an armistice; and then there may be unrequited attachments. There may be mental ailments to which not the dull, and gross, and stupid, but those most distinguished by sensibility and talent, are most subject, and from which the latter most suffer, which are "past all surgery," and for which physic proffers no remedy. The quack indeed proffers it, and some are found to sell themselves to the devil of empiricism; and well are their frailties turned to profit, but their cure is to be attained,

"Non tali auxilio nec defensoribus istis."

The patient can no otherwise recover than by returning—whatever be the path he takes—to the regions of truth, nature, and common sense. It is "metaphysical aid" that he needs. The complications of disease, mental and bodily, which afflict man, the most complex of animals, require the most extensive and profound scholarship to read aright. It would be as irrational for patients suffering from a wounded mind to consult the fortune-teller as the quack-salver; those are to be consulted to most advantage to whom, not the secrets of medicine only, but of human nature lie open, and to whom just and kindly views of the subject are familiar—who are capable, not of giving advice only, but of truly sympathizing with every species of human infirmity and human affliction.

The effect of quack treatises and advertisements is most pernicious, most calculated to put unnatural fears into young minds, and to tend to no end except that which is sought, to extract money from their purses, and induce them to swallow some pecks of pills and gallons of liquids, under the name of physic, as filthy, but not as harmless, as so much hogwash. There can be no return to a healthful and true condition of mind through such means, under such auspices. It is, in ninety-nine cases in every hundred, the mind alone which is at fault. There is a growing want among civilized communities of marked individuality. We are rather machines than men: the state of society has become too complex and artificial. The thievish conventionalities of life engulf all natural goodness, extinguish all natural passion. We are all the victims, more or less, of a state of systematized unhappiness. We lose the faculty of seeing things as they are. Unseemly visions of things which are not group themselves around us. All society is an aggregate of fallacies. We become accustomed to these, till truth dazzles and pains us. It is no marvel that the master passion should participate in the obliquities of this universal malady. We make ourselves ill, or submit to be made ill, in mind and body; we swallow physic; and we call this civilization. It is truly shocking to think that there are persons besotted enough to believe that swallowing any sort or amount of physic can cure ailments which, in so far as they may be said to exist at all, are wholly of a metaphysical character. It may be said that a man may as well perpetrate one sort of folly as another, and it is in the nature of all follies to aid him in passing away his time; at the same time it must be allowed, that physic-swallowing is a very dirty description of amusement, and that it would be well for the "dove's wings" of the spirit "blackened among the advertising apothecaries' pots" to resume the beauty and purity of their plumage. The outlay in advertisements of quack treatment and remedies for ailments, for the most part wholly imaginary, is tremendous; it would not be kept up did it not prove a lucrative investment of capital, by which multitudes of persons are being continually fed, and at whose shrine multitudes of victims are continually immolated. And what is the truth, looking at the subject largely? That John Bull is a born blockhead and congenital hypochondriac, whose maladies, of a sexual kind especially, are assuredly deep-seated enough, but deep-seated only in his imagination; and that these advertising vagabonds are making a dupe of him and a market of the infirmities with which their own writings inoculate his mind; that he is a gentleman, in short, whose loins, if they have any peculiar failing, labour under the misfortune of being only too prolific. If, among the component atoms of the said John Bull, there are exceptions, these are few in number: the great mass of exceptions being made out to be such by commentaries upon symptoms, most of which, whether in the human or brute creation, are merely *propria quæ maribus.* What must foreigners, or persons of other ages and climes, infer from the columns of our daily papers? That we are a nation so imbecile as to be perpetually on the verge of extinction—that the being and name of Englishman would, in threescore years and ten, cease from the face of the earth, were it not for the medicines which we are obliged continually to swallow down wholesale, in order to qualify us for the perpetuation of our species. The inference would not be an unfair one; the whole question clearly admits of such a *reductio ad absurdum.* It would seem as if, in more respects than this now under discussion, the English were a nation afraid of shadows at home, and beating everything before them abroad. Seriously speaking, among all got-up, and encouraged, and extensively prevalent modern delusions, no monster so demands to be smitten down as this; and that it does prevail extensively enough to constitute a lucrative object of speculation, the sums expended in advertisements for the cure of John Bull (poor man!) of his incapacities pretty clearly evidence. I am quite certain that the case here reported was not spermatorrhea, nor any species of bodily disease whatever, but that it was mental, and a case of monomania, generated by circumstance and accident, as operating upon a sensitive and impressible temperament. It was an extreme case of *mauvaise honte,* the commonest of all English mental maladies. A famous market is being made of it by a low class of adventurers; and a most dirty and dishonest source of traffic it is. It is a common spectacle enough to see poor John Bull fallen among thieves, and these thieves mostly under the garb of good Samaritans.

In recent works, masturbation, venereal excesses, gonorrheal discharges, and inflamed urethra, have been named as causes,—spermatorrhea, as the effect. This theory has much plausibility and verisimilitude about it: it is, nevertheless, a not uncommon thing to meet with facts which go against all likelihood, and with facts which do not concur with likelihood. It is my persuasion that spermatorrhea is, in point of fact, a disease *per se,* with which the causes named, as such, have nothing necessarily to do; that it is characterized by more or less incapacity for not mutually satisfactory sexual connexion only, but for masturbation. There shall be an ascertained case of spermatorrhea: well, any man, patient or not patient, ill or well, when closely questioned, will always have something genital to confess, if he chooses to confess it; but that something is not to be rashly set down as the cause of spermatorrhea. It must be recollected that there is *always* something that might be owned respecting passions repressed, encouraged, gratified, or subdued. There is always something. It would be quitting physiology, and trespassing upon the borders of psychology, to remark with what scorn a strong, especially if a virtuous, individual attachment regards any gratification of the passions, except with the object of such attachment. Religion and morality may and do supply nobler motives for the subjugation of the passions; but, as human nature is constituted, they supply none so generally effectual. But it may be said, indeed, with regard to such an attachment, that religion and morality cannot but co-exist, and co-operate with its own special and pure impulses. The victims of quacks and of quack advertisements should know these things —should act up to them. Theirs are, for the most part, not cases for swallowing physic, little or much; their complaints are such as rather require ethical than therapeutic treatment. The success of quack advertisements of a certain description is no matter of trifling comment— there is a great deal in it. There are important inferences to be deduced from it. Were not the number of those whom such advertisements influence great, the projectors of them could not afford to pay for their incessant insertion. This leads to a well-founded surmise, that among minor cases of monomania, that even among promiscuously-observed cases of absolute alienation of mind, there must be very many which have, so to speak, a genital origin. It is not inferred that they originate in genital disease, but in the human structure, in so much and so far as it is genital. This inference leads to a wide range of investigation, embracing the points of affinity between what may be termed genital physiology and psychology; a most difficult and dangerous subject, on which a word uttered awry might do much moral evil. There are also minor cases of monomania, arising, not from genital organization, but from actual genital disease. A man shall have gonorrhea. If he be ignorant and timid, he shall think that his very life, or if not his life, his virile powers, are, in the visible shape of the blenorrheal discharge, actually oozing away. Now we all know that the discharge, as a discharge, forms no appreciable tax upon a good constitution, and that the constitutional irritation set up is seldom of any serious consequence. We have here the example of a minor kind of monomania of frequent occurrence. It is indiscribable what terrible superstructures a diseased imagination will raise. The reverse of this will sometimes happen. A man shall have a chancre. He fells no pain worth calling pain: he sees no danger. He neglects himself, and suffers severely from his temerity and inattention. In the former case of a man terrifying himself into fatuity, or into the grave, with shadows of his own raising,—what have we to do? Certainly to pay such overt attentions as shall prevent his flying off at a tangent, and getting into worse hands; but not to attend to this point only, and to the prescription of the remedies really needed, but also to soothe and to unfrighten him. We have to deal with him, not as a sufferer from disease only, but from monomania; the latter complaint being often the more difficult of treatment of the two, as it is sometimes of the two the more serious.

In conclusion, it is obvious that genital physiology and genital pathology are nearly allied to psychology. There are few cases of insanity in which there is not to be remarked simulation and dissimulation. Craft is shown both in concealment and reserve, in affectation and in some species or other of ostentation. Could we get at the truth, we should often find the dignity attendant upon devotional mania fade away from our sight, and mental disease so marked be traced beneath this surface to some obliquitous direction taken by what has been termed the master passion. The number of those who approach the vestibule —who pass the threshold—of insanity, through its labyrinthine windings is greater than is commonly imagined. There may exist not simulation and dissimulation so much as blindness and self-delusion on the part of the victims as to the *causa causationis*. It is not to be denied that even a praiseworthy and virtuous repression of the passions may in some instances generate cerebral disorder and disease, and terminate in the establishment of insanity. But better that the mind should thus, before the body dies, pass away "walking in its uprightness," than that its possessor should lead a lengthened life of intellectual sanity but moral turpitude, especially considering that not far off from the most long-lived of our race is that world to come in which all sufferings in a good cause will be amply compensated, and all wrongs will be for once and for ever righted. We can take no accurate, no enlarged view of persons and things which is taken irrespectively of the world to come. Those who repudiate religion, because cases of religious mania occur, should, in order to be consistent, consent to a like dereliction of humanity out of consideration for such cases of insanity as are of a genital origin. There is a couplet in which it is said of a person whose character is portrayed—

> "Each passion that invades the human breast
> Had troubled his brain's clearness and heart's rest."

*That* is the thing to give a man a knowledge of all sanity and all insanity. If he knows nothing either of the influences of religion, or of the passions, he knows little of human nature: if he knows only one of the two, he knows but half of what he ought to know. The knowledge which life and experience supply exceeds in value what can be learned from books. Too much of such knowledge, a mind too much enlarged, too well informed,—cannot be possessed by him upon whom devolves the treatment of the insane. The habitudes and smaller capabilities of a keeper rendered obtuse to the spectacle of mental alienation, are less requisite to the management of the insane than the supervision and superintendence over all of a liberal and enlightened mind The same qualities are indeed necessary daily and hourly in the usual routine of all medical practice. Minds not insane, but prostrated by disease, require address and management, and to manage them their condition must be understood. Enough has been said in the statement of the foregoing case to show that there are no misprisions imaginable to which the mind of man is not liable; and that the most favourable interpretation that can be put upon a doubtful case of human conduct will in some instances be the most correct.

[The above case is from the pen of a professional gentleman of great respectability, personally known to us. It is necessary to say that we do not acquiesce in all the remarks made by the author, particularly in those portions in which he refers to the ethical question—whether we are not justified, under certain circumstances, in deviating from what are considered by the Divine Author of religion as stringent moral obligations in order to avoid the commission of offences most destructive to our mental and physical well-being? We maintain that there can be no justification for the violation of the law of God. We are bound to obey implicitly, and even blindly, the mandate of the Supreme Being, *malgré* the consequences. Great apparent suffering may be the result of an implicit subjection to the literal command; but it is consolatory to reflect, that He who issues the mandate has promised "to temper the wind to the shorn lamb." Whilst on the subject of spermatorrhœa, it was our intention to have referred to the interesting work of *Lallemand*, so ably translated and edited by G. K. J. Macdougall, Esq. We must defer this pleasure to another occasion.—Editor.]

